# Assessing the physical activity training needs and preferences of community health workers in South Africa

**DOI:** 10.1186/s12889-025-21352-z

**Published:** 2025-01-10

**Authors:** Mark Stoutenberg, Blanca S. Noriega Esquives, Ruth G. St Fleur, Susanna S. Koen, Estelle D. Watson, Francia G. Portacio, Georgia Torres

**Affiliations:** 1 Department of Sport and Exercise Sciences, Durham University, Durham, England; 2 Department of Exercise Science & Sports Medicine, University of Witwatersrand, Johannesburg, Gauteng, South Africa; 3 Department of Public Health Sciences, Miller School of Medicine, University of Miami, Miami, USA; 4 Department of Epidemiology, School of Public Health, Brown University, Providence, USA; 5 Netcare Rehabilitation Hospital, Johannesburg, South Africa; 6 INMED South Africa, Johannesburg, South Africa; 7 Department of Exercise Science, Faculty of Science, University of Auckland, Auckland, New Zealand; 8 Perelman School of Medicine, University of Pennsylvania, Philadelphia, PA USA

**Keywords:** Community health workers, Desires, Non-communicable disease, Physical activity, Preferences, Training

## Abstract

**Background:**

Emerging work highlights the potential of community health workers (CHWs) to promote physical activity (PA) as a part of their role in preventing and managing non-communicable diseases. However, little is known about CHW preferences and desires towards receiving PA training.

**Methods:**

Community health promoters (CHPs), a type of CHWs in South Africa, from seven health districts in Johannesburg participated in a day-long in-service training on PA and healthy eating. Prior to the training, CHPs completed a 22-item questionnaire assessing their PA attitudes, beliefs, past promotion efforts, and previous PA training. CHPs were divided into small focus groups to discuss their roles, amount and type of PA training they had received, perceived PA knowledge, and desire for future PA training. A mixed methods approach triangulated data from the questionnaire and the focus groups.

**Results:**

Seventy-six CHPs attended the in-service training, completed the questionnaire, and participated in one of ten focus groups. CHPs were 38.5 (± 9.2) years of age, 58.7% were male, and 85.3% had > 2 years of experience. Nearly all felt PA was beneficial for health (89.5%) and that promoting it was a part of their job (85.3%). Most CHPs felt that they had sufficient PA knowledge (93.1%) and reported knowing global PA guidelines (90.5%). However, only 10.5% correctly identified the guidelines for aerobic activity or muscle-strengthening, and none correctly identified both. CHPs expressed great interest in receiving additional PA training (98.5%) and applying it in their work (97.1%). Five themes emerged from the focus groups: (1) roles and responsibilities in health promotion and disease prevention; (2) receiving ongoing training to enhance knowledge and skills; (3) increasing their PA training to foster healthier lifestyles in their communities; (4) desired structure of future PA trainings; and (5) strategies for applying the PA training.

**Conclusions:**

This work provides further evidence for the potential of CHWs to take on a greater role in disease prevention, such as promoting PA. However, future research needs to explore strategies to: provide regular, ongoing PA training, enhance internal team dynamics, and integrate PA promotion as a regular part of their clinical responsibilities and community outreach.

**Supplementary Information:**

The online version contains supplementary material available at 10.1186/s12889-025-21352-z.

## Background

Over the past three decades, we have witnessed a global epidemiological transition - a changing pattern of morbidity and mortality from infectious to non-communicable diseases (NCDs), with NCDs account for 57% of all premature deaths. Individuals in 88% of countries worldwide having a higher probability of premature death from NCDs than from communicable, maternal, perinatal and nutritional conditions combined [1, 2]. The increasing burden of NCDs and related mortality is a major public health issue, particularly in low- and middle-income countries (LMICs) where 78% of all deaths, and 86% of premature deaths, are attributed to NCDs [3]. In sub-Saharan Africa, disability-adjusted life years lost due to NCDs increased 67% between 1990 and 2017 [4].

In 2017, the World Health Organization updated their 16 best buys for the prevention of premature death, highlighting the importance of addressing four leading behavioural risk factors - tobacco and alcohol use, unhealthy diets, and physical inactivity [5]. Maintaining a healthy lifestyle is associated with 9.9 and 9.4 additional years without chronic disease in men and women, respectively, with physical activity (PA) identified as one of four lifestyle habits associated with the longest disease-free life span [6]. However, the prevalence of insufficient PA (< 150 min per week) has increased to 27.5% of all adults globally, with 38.2% of the South African adult population classified as insufficiently active [7].

As part of a comprehensive approach to addressing NCDs, new strategies for increasing PA levels are needed. One strategy, highlighted in multiple calls to action, is through the engagement of the healthcare sector [8–10]. The effectiveness of PA promotion embedded within health systems is well documented, but primarily in high income countries [11–13]. In LMICs, where access to and funding for primary healthcare is often inadequate, community health workers (CHWs) fill an important role addressing shortfalls in human resources at the intersection of individuals, marginalised communities, and health systems [14, 15]. Emerging work highlights the potential of CHWs to promote PA as a function of their outreach responsibilities through informative approaches, behavioural counselling, and leading programmes [16].

CHWs have been a vital part of healthcare service delivery as far back as the 1930s [14]. The Millennium Development Goals (released in the late 1990s) generated renewed interest and reinvestment in CHW programmes [17]. Between 2005 and 2014, there was nearly a seven-fold increase in publications involving CHWs, half from Africa [18]. However, only 4.5% of these articles focused on NCDs and gaps remain in engaging CHWs in NCD prevention efforts [19]. In Uganda, a majority of CHWs were aware of and involved with NCD-related activities, but nearly 60% reported lacking knowledge and only 20.6% had actively referred patients to health resources [20]. The South African Ministry of Health stated that addressing NCDs and their risk factors should be fully integrated into health systems [21, 22]. A foundation of the proposed integrated care model is an expansion of primary care in community settings, led by trained CHWs conducting health and wellness outreach [23].

With the growing roles of CHWs in LMICs, global standards provide generalised guidance for CHW training [24]. In South Africa, a three-phase approach for CHW training was developed with the second phase, initiated in 2014, consisting of NCD training [25]. De-centralised systems were established at the district/sub-district levels to disseminate the training, but a national appraisal found the training inadequate due to poor planning and little to no continuing education [25]. In general, there is a lack of research exploring NCD-related training and PA promotion for CHWs. Considering the significant roles of CHWs in NCD prevention, an exploration of their training desires in carrying out these functions is warranted. Therefore, this study aimed to investigate CHW needs and preferences for receiving PA training so that PA promotion efforts could be further incorporated as a regular part of their work responsibilities.

## Methods

This study was conducted over three days in July 2019 and involved community health promoters (CHPs) from the seven health districts in the City of Johannesburg (CoJ), who participated in a full day of in-service training on PA and healthy eating. CHPs from 2 to 3 health districts attended the training each day to balance the number of individuals present at any one time. The CHPs completed a questionnaire and participated in focus groups prior to the training. All study procedures and trainings were repeated over each of the three days.

### Setting

This study was conducted in the CoJ, one of five districts in the Gauteng Province of South Africa, which is home to more than 4.4 million people. The in-service training was held at the Social Development Office in Orlando, Soweto, a township of the CoJ. The office consisted of indoor space for presentations, a courtyard for PA demonstrations, and an aquaponics site, developed in partnership between INMED South Africa [26], a non-profit organization, and the CoJ as a part of an adaptive agriculture program to ensure a local, sustainable food and opportunities for income generation in the local community.

### Participants

The CHP training was organized in partnership with the CoJ Department of Health. The CHPs were paid employees of the CoJ, many had at least a high school diploma, and they filled a role similar to that of traditional CHWs. In the CoJ, CHPs are often hired into more permanent positions by the government, while CHW roles tend to be temporary or consist of contract work, although the actual work responsibilities between the two positions tend to be relatively similar. One CHP is typically assigned to each community health centre to work with patients both in the clinic (e.g., support groups) and in the local community (e.g., hosting community health events).

The CoJ Department of Health provided transportation for all CHPs to the training site. The training was considered as continuing education and a mandatory part of their job responsibilities. Once onsite, the study investigators guided the CHPs through the informed consent process and emphasized that participation in the research activities was optional and would not impact their participation in the training activities. Approval for the study and all materials was granted by the Human Research Ethics Committee (Medical) at Wits University (certificate no. M170273).

### Questionnaires

After the informed consent process, the CHPs completed a 22-item, paper-based questionnaire (Additional File 1) that was designed by the research team specifically to gather descriptive information on the CHPs background training and experiences. The questionnaire captured basic demographic information, assessed knowledge of PA guidelines, PA attitudes and beliefs, PA promotion efforts, awareness of existing PA programmes/classes in their community, receipt of previous PA training, and the level of interest, desired format, and specific topics to cover in future trainings.

### Focus groups

CHPs were then divided into smaller focus groups (FGs). Three (*n* = 21 participants), four (*n* = 31), and three (*n* = 24) FGs were conducted across the three days. Each focus group was led by a research team member, with the study principal investigator providing general oversight. At the end of each day, the research team convened to debrief on their experiences and ensure consistency in leading the FGs on subsequent days.

A semi-structured interview guide (Additional File 2) was developed for the FGs. The guide included open-ended questions on how the CHPs viewed their roles and responsibilities, any previous training they had received, skills needed to be a CHP, any previous PA training they had received, self-perceived knowledge and skills to provide PA information to patients/community members, and how they would ideally like to receive PA training in the future. Eight of the 10 FG sessions lasted 45–50 min, while two were abbreviated (25–30 min) for late-arriving CHPs on the first and third days. All FGs were conducted in English, digitally recorded, and later transcribed verbatim by a research team member.

### Data analysis

All questionnaire data were downloaded to an electronic spreadsheet and reviewed for consistency. All completed questionnaires were included in the final analysis regardless of missing data; consequently, the number of responses for each item varies. Estimated means and standard deviations were produced for continuous variables, while counts and percentages were calculated for categorical responses. Frequency analyses were conducted for items querying CHPs about their PA attitudes and beliefs and their PA-related interactions with community members/patients. Theoretical thematic analyses were used to analyse open-ended questionnaire responses.

This study employed thematic analysis grounded in an experiential orientation to examine how our participants interpreted and engaged with the phenomena of interest (e.g. promoting PA as a part of their work responsibilities). A combined inductive and deductive approach was used to analyze the FG data, centering participant perspectives to reflect their experiences. First, authors read a subset of transcripts to develop an initial codebook. Raters (BNE & RGS) then coded one transcript together to refine the codebook. The research team met regularly to discuss codebook changes, verify that codes were applied systematically, and reach consensus on discrepant ratings. Another transcript was selected to assess the percentage of agreement between raters. Initially, the two raters agreed on 81% of the independently coded data, with 100% consensus reached through discussion. Coded text was entered into Dedoose (version 7.0.23) to perform analysis and extract coded participant responses. Codes were sorted into categories derived from the interview guide. Finally, the research team reviewed all codes and categories several times to identify meaningful themes. This systematic approach was taken to ensure trustworthiness of the data.

## Results

A total of 76 CHPs attended the in-person trainings, completed the questionnaire, and participated in one of ten FGs.

### Questionnaire data

Basic demographic characteristics of the CHPs are provided in Table 1. The CHPs averaged 38.5 (± 9.2) years of age, 58.7% were male, and 85.3% reported having ≥ 2 years of work experience. Fifty-seven CHPs (77.0%) felt they were either skilled (36.5%) or highly skilled (40.5%) at their jobs.

**Table 1:** Community Health promoter (CHP) demographic characteristics

	All CHPs
Age in Years (mean, SD)	38.5 (9.2)
Sex (n, %) Male Female	44 (58.7) 31 (41.3)
Years of Experience (n, %) <2 years 2–5 years 5–10 years 10–20 years >20 years	11 (14.7) 27 (36.0) 18 (24.0) 14 (18.7) 5 (6.7)
Race (n, %) African Coloured	73 (97.3) 2 (2.7)

* 75 of 76 CHPs responded to each of these items. CHP: community health promoters; SD: standard deviation

CHP PA attitudes and beliefs are provided in Table 2. Nearly all CHPs strongly agreed that PA was beneficial for health (89.5%), that promoting PA was a part of their job (85.3%), and that they could help community members increase their PA levels (agreed, 48.0%; strongly agreed, 49.3%). Most CHPs strongly agreed (35.6%) or agreed (57.5%) that they had sufficient PA knowledge and reported being familiar with global PA guidelines for adults [27]; however, few CHPs correctly identified aerobic activity (10.5%) or muscle-strengthening (10.5%) guidelines, and none correctly identified both.

**Table 2:** Community Health promoter (CHP) attitudes and beliefs towards physical activity (PA)

	Strongly Agree	Agree	Disagree	Strongly Disagree
PA is beneficial for health* (n, %)	68 (89.5)	6 (7.9)	0 (0)	2 (2.6)
Any amount of PA is beneficial^^ (n, %)	36 (48.6)	29 (39.2)	7 (9.5)	2 (2.7)
Promoting PA is part of the job for CHPs** (n, %)	64 (85.3)	11 (14.7)	0 (0)	0 (0)
I have sufficient knowledge about PA^^ (n, %)	26 (35.6)	42 (57.5)	4 (5.5)	1 (1.4)
I can help community members increase their PA levels** (n, %)	37 (49.3)	36 (48.0)	0 (0)	2 (2.7)
I regularly advise community members about PA* (n, %)	59 (77.6)	15 (19.7)	1 (1.3)	1 (1.3)

* 76 respondents, ** 75 respondents, ^ 74 respondents, ^^ 73 respondents

CHP: community health promoters; PA: physical activity

CHPs reported varying levels of patient interest in PA (Table 3). Forty-four (59.6%) reported that 50% or more of their community members/patients asked about PA, while 50.7% of CHPs reported recommending PA to all patients. CHPs commonly recommended walking (*n* = 28), running (*n* = 18), aerobics (*n* = 17), and muscle strengthening (*n* = 12) activities. Nearly all CHPs (87.7%) reported being aware of local PA programmes, referring patients to local aerobic activities (*n* = 29), walking/running groups (*n* = 12), community gyms (*n* = 10), local clubs/support groups (*n* = 10), or specific activities for older adults (*n* = 8). Finally, 57.8% and 29.7% of CHPs always or often recommended local PA programmes to their patients, respectively. The most common barriers to recommending PA included patient illness or medical condition (*n* = 12), physical disability (*n* = 2), or going against a physician recommendation (*n* = 2).

**Table 3:** Community Health promoter (CHP) physical activity-related interactions with community members/patients

Percent of Patients that asked CHPs about PA	Number of CHPs (*n*, %)^*^
0	2 (2.7)
25	28 (37.8)
50	21 (28.4)
75	16 (21.6)
100	7 (9.6)

**Percent of Patients to whom CHPs recommended PA**	**Number of CHPs** **(n**, %)^**^
0	0 (0)
25	6 (8.0)
50	14 (18.7)
75	17 (22.7)
100	38 (50.7)

* = 75 responses; ** = 74 responses; PA: physical activity. CHP: community health promoters; PA: physical activity

Slightly more than half of the CHPs (52.1%) reported receiving prior PA training. Nearly all expressed that they would be interested/very interested (96.5%) in receiving more PA training and that they would be likely/extremely likely (97.1%) to apply this training in their work. The CHPs were most interested in learning how to promote PA for specific populations, such as older adults and pregnant women (*n* = 14), the benefits of PA (*n* = 9), specific exercises for their patients (*n* = 9), and the basics to developing exercise prescriptions (*n* = 8).

### Focus Groups

Ten FGs, involving 76 CHPs, were held over three consecutive days. Five main themes emerged from the FGs (see Additional File 3 for specific quotes).

1. *CHP roles and responsibilities in health promotion and disease prevention*

The CHPs self-identified as frontline health workers serving as a bridge between health clinics and the community. Their duties included helping patients navigate the healthcare system, implementing national health policies, and advocating for their communities. Most duties were carried out in health facilities (e.g., guiding patients on obtaining referrals), with some activities carried out in community settings (e.g., community outreach, immunization efforts). The CHPs perceived themselves as health educators and health promoters and that primary prevention was their main duty. Educating the community about disease prevention and health promotion provided them with a high degree of job satisfaction. They highly valued delivering health education, organizing primary prevention activities, and mobilizing the community. They also provided secondary and tertiary prevention by conducting infectious disease screenings (e.g., human immunodeficiency virus [HIV], tuberculosis) and promoting chronic disease self-management by educating patients on the benefits of treatment adherence and regular follow-up visits.

Many CHPs believed they possessed good communication skills, which included being comfortable speaking in public, communicating using clear and engaging language, providing counselling, and facilitating group discussions. They felt confident in their ability to mobilise the community and organise health promotion activities. However, they also felt that their health promotion role was misunderstood by nurses and other healthcare workers. They expressed unhappiness with the facility managers’ views of their role, who expected them to spend most of their time in the clinic assisting staff rather than being in the community. The CHPs also described several other barriers to performing their duties, including a lack of resources, a lack of patient interest in health education, and patients expecting to receive rewards for attending health promotion activities. Due to a lack of resources, CHPs needed to be resourceful in conducting their health promotion activities, such as finding sponsors to provide food and drinks for participants.

2. *Ongoing training to maintain skills and knowledge*

Overall, CHPs felt adequately prepared for their job, but acknowledged that additional skill-based training was needed. CHPs commented on needing to improve their planning skills, having the capacity to develop work plans, and learning how to delegate responsibilities to excel in their jobs. They also expressed interest in receiving more training to improve their communication skills in working with people from different age- and cultural groups. Most CHPs felt confident in their knowledge of health promotion and disease prevention, but felt it was important to be up to date with health information as patients had easy access to information (i.e., via smartphones). Several CHPs mentioned regularly seeking out health information online because they did not receive sufficient training. Although some CHPs participated in multiple training sessions each year, trainings were not usually planned in advance. Topics included healthy lifestyle behaviours (22/67 topics mentioned, including nutrition and PA), infectious diseases (16/67, including HIV and tuberculosis), chronic diseases (16/67, including diabetes and hypertension), and treatment adherence (5/67). Most trainings were described as ‘informal’ consisting of unstructured didactic sessions, with no examinations or formal accreditation. The duration of the training was usually one day, with more formal trainings lasting up to five days. Although CHPs believed that the trainings provided them with updated and relevant information, they were not seen as sufficient for career advancement.

3. *Need for PA training to foster healthy lifestyles in their communities*

CHPs recognized the importance of lifestyle behaviours, such as PA and healthy eating, for health promotion and chronic disease risk reduction. They believed that healthy lifestyle behaviours should be part of a chronic disease management plan. CHPs stated that when patients complained about a lack of time, they encouraged them to see PA less formally and as a part of their everyday life. Thus, CHPs spent most of their time teaching people how to incorporate healthy lifestyle behaviours into their daily routine (e.g., riding a bike to work, exercising at home without equipment). They also reported educating people on the benefits of PA beyond weight loss, such as improving sleeping patterns and decreasing stress levels.

The CHPs also highlighted the importance of being role models for healthy lifestyle behaviours. Several CHPs mentioned that in order to teach healthy lifestyle behaviours to the community, they needed to change their own behaviours first. They mentioned that the community liked seeing them doing things they advised others to do (e.g., daily exercise, ordering healthy foods). CHPs noted several socio-economic barriers that community members faced in adopting healthier lifestyles (e.g., poverty, neighbourhood safety, lack of access to recreational facilities or healthy foods). Because of these barriers, CHPs often did not see expected results among their local community members.

Despite having a good grasp on PA-related knowledge and the importance of engaging in PA, CHPs pointed to an inability to demonstrate and practice exercises with patients due to a lack of practical training. They discussed having a lack of knowledge in tailoring activities to specific subgroups, such as people with physical limitations (e.g., back issues, heart problems). Several CHPs expressed not having adequate knowledge when it came to promoting PA among pregnant women, such as the appropriate intensity and types of exercises. Other CHPs expressed an interest in learning more about age-appropriate exercises for older adults. Further, CHPs pointed out a need to learn how to adapt PA (e.g., types of activities) for use in different settings and with vulnerable community members. Given these gaps in PA knowledge and skills, CHPs expressed great interest in receiving more frequent, practical training.

4. *Perceptions of previous PA training and suggestions for future trainings*

When discussing previous PA training, CHPs reported receiving little to no formal training. Three CHPs reported receiving PA training at school. Other CHPs reported receiving PA training the year before through a local university. CHPs that attended this training recalled receiving helpful population-specific PA information, such as exercises for pregnant women and older adults, and reported being able to diffuse the information to the community. A shortcoming of the training included a focus on theory and a lack of applied exercises. CHPs stated that the brevity of the training (i.e., crammed into one day) made it difficult for them to fully master the amount of information that was being provided and that there was no follow-up.

Given the informal nature of previous trainings, CHPs expressed the need for structured PA trainings, planned in advance, and delivered by experienced professionals (e.g., biokineticists, CoJ Johannesburg Sport and Recreation department staff). There was a desire for PA trainings to span several days over a week, with some suggesting a week as the ideal duration, and occur throughout the year to provide ongoing support. CHPs recalled being challenged by patients who often had a negative attitude towards engaging in PA, as a result of mistrust of their ability to provide PA guidance. Consequently, CHPs expressed a need for post-training assessments that tested their PA knowledge and skills, qualifying them for accreditation. This would enhance their legitimacy in the eyes of the community, increase trust, and improve their health education and PA promotion efforts.

When discussing future PA trainings, CHPs expressed a preference for more specialized content. They desired trainings tailored around specific medical conditions, as well as the needs of different age groups. CHPs wanted to receive specialized PA training for individuals with chronic conditions (e.g., asthma) and those with physical disabilities. Given their lack of skill demonstrating exercises, CHPs opted for an equal focus on both theoretical and practical aspects of PA. CHPs also desired training to help patients perform exercises in different settings (e.g., during clinic visits), with and without equipment. CHPs also suggested that the individuals providing the training visit their clinics to see the context/setting in which they worked. A better understanding of their clinic setting would help customise the training to available resources. Lastly, CHPs saw the PA training sessions as an opportunity to improve clinic staff support for their roles and capabilities. Including nurses in the trainings could generate more support for CHPs to provide health education with patients at the clinic and allow them to better integrate PA promotion into their work.

5. *Application of PA training*

To effectively promote PA to patients who visited their clinics, CHPs expressed the need for equipment, such as dumbbells or elastic bands. They also mentioned the need to leave the trainings with relevant visual aids that would not only serve as reminders, but also help guide their work with the patients, such as manuals that they could use to refresh their memory. The CHPs also wanted pamphlets or booklets with drawings that could be shared with patients during their PA promotion efforts. Additionally, numerous CHPs voiced interest in having video ‘clips’ of exercises, posters for the clinic, and having projectors that could be used during onsite work with their patients.

## Discussion

The 2004 National Department of Health CHW policy framework encouraged provincial departments to establish generalist CHW programmes in disadvantaged communities throughout South Africa [28]. An important part of this framework was unifying CHW training standards to address health promotion, given the dramatic increase in NCDs over the past two decades. However, major gaps remain in establishing standardised training programmes in LMICs and little known about providing CHWs with the knowledge and skills to address NCDs in their community, including PA promotion efforts. We present our findings in which our CHPs describe their roles and responsibilities, previous general and PA-specific training received, involving clinic staff in training efforts, providing credentialing/certification, and applying PA training with patients and community members.

### Roles, responsibilities, and general training of CHPs

There is a growing awareness of the important role CHWs can play in disease prevention and health promotion [29–31] and the effectiveness of CHW-led NCD prevention efforts [19, 32]. A main finding of our work was that almost all CHPs felt that they played an important role in NCD prevention and control and viewed themselves as prevention leaders, proactively promoting health. CHPs felt that promoting PA was a part of their jobs and nearly all (97.3%) reported regularly advising community members. This is similar to CHWs in Uganda who felt that they were an important part of NCD prevention and control [20, 33]. However, many CHWs still operate within more traditional, narrowly-defined roles and scope of work (e.g., programmes targeting infectious disease and maternal health) [20, 34, 35]. In Uganda, community members expressed concerns about the ability of CHWs to deliver NCD services [20]. In the Northern Cape of South Africa, CHWs felt their first mandate was to carry out screenings for pregnancy, palliative and wound care, pregnancy registration, medication adherence, and checking immunization status of babies [34]. In Ethiopia, nearly half of the CHWs had unfavorable attitudes (i.e., lack of motivation) towards promoting healthy lifestyles [35].

Multiple studies point to the importance of providing high quality, culturally appropriate training to ensure that CHWs have the skills necessary to carry out their jobs [36]. In our study, CHPs study expressed a desire for ongoing training and continuing education related to all aspects of their job, as they often did not have adequate information on a topic and had to resort to finding information. When they received continuing education, it was often informal, with no advance notice, and crammed into an intensive session with no follow up. Previous work demonstrates that regular education and field-based refresher trainings increase job satisfaction, knowledge, skill development, and overall competence [37]. Trainings need to be conducted by professional staff, should be ongoing and iterative, and use a mix of knowledge- and skill-based approaches, field observation, and continuous supervision [38–41].

### PA-specific training recommendations

The CHPs in this study reported receiving little or no PA training other than a single day the previous year. Those who attended commented that it was theoretical, lacked direct application, and included no follow-up training or supervision. While 93.1% of CHPs reported having sufficient knowledge about PA, none were able to correctly identify guidelines for aerobic activity and muscle-strengthening. Similarly, 97% of CHWs in Brazil reported a desire for more information on PA, with only 3.6% correctly identifying WHO PA guidelines [42]. Existing literature continually points to inadequate training and preparation of CHWs, particularly as it relates to NCD prevention and treatment [30, 43, 44]. Equally important as the initial training is establishing an ongoing process of continuing education, a need noted by several CHPs. Musoke et al. found that CHWs who received additional training or refreshers were 12 times more likely to be classified as ‘high performers’ [45].

CHPs felt that their training activities needed to be spaced out over more than just a single session or day. A scoping review estimated that CHWs received, on an average, only six hours of total PA training [46], far less time than is typically dedicated to other health conditions, such as infectious disease and maternal and child health (e.g., both twice monthly over a year) [47, 48]. Delivering a limited amount of PA training resulted in only small increases in CHW knowledge and no future improvements in their own PA levels or body mass index [49, 50]. CHPs expressed a need for more applied training that they could incorporate with patients in the clinic (e.g., in waiting rooms) and community settings. Providing experiential learning experiences is a relatively common training technique used in CHW training curricula [51]. The CHPs also desired more training with specific populations, such as pregnant women and older adults. However, providing this in-depth training may increase training-associated costs, minimizing the cost-effectiveness of using CHWs in PA promotion and NCD prevention efforts [51]. Other alternatives, such as providing CHWs with a basic level of training to conduct PA screening, providing brief counseling, and referring patients/community members to more specialized professionals and/or programmes, should be explored.

### Enhancing the application of the PA training

Another key point that emerged from our work is that CHPs desired materials to remember the training and better promote PA with their patients/community members. CHPs expressed wanting a way to access training information in the future and suggested having manuals and video clips as a reminder of information learned, a strategy that has been used in PA training courses in high income countries [46] and with other health conditions in LMICs [41, 52, 53]. Similarly, CHPs discussed having a lack of supplies (e.g., dumbbells, elastic bands) that negatively impacted their ability to assist patients/community members and that having more equipment available to them would aid in the PA promotion efforts.

### Support and involvement of clinic staff

CHPs in the current study felt a disconnect between their knowledge, skills, and the work they could perform (such as promoting PA), and the perceptions of their supervisors (e.g., nursing staff). CHPs described the lack of support from their clinic staff as demotivating. While a study in rural South Africa reported a positive relationship between CHWs and their nursing staff [34], most research describes CHWs as feeling underutilized, under-appreciated, and underpaid [40, 43, 54]. In the redesign of South African primary care health teams, a mid-level nurse (or outreach team leader) was envisioned as supervising CHWs and spending 70% of their time in the field [55]. However, competing clinical demands have pulled the nurses, and often the CHWs, back into more clinical-facing roles. Interprofessional challenges may also arise from hierarchical work structures, displays of professional superiority, and a lack of clear roles [37, 43]. When CHWs receive supportive supervision, the quality of their work improves (e.g., higher fidelity scores and task performance) [56]. In Thailand, staff appreciation of CHW contributions, regular meetings between staff and CHWs, involvement of CHWs in planning and implementation of health programmes, and encouragement by higher-level health officials, were identified as best practices for supporting CHW efforts [57]. The performance of CHWs, regardless of their level of training and expertise, is highly associated with organizational support and the extent to which they are understood by clinic staff [56].

Thus, building lasting and sustainable relationships within health teams based on trust, recognition, and empowering CHWs to fulfill their roles, are critically important. Several CHPs discussed the need to include supervisors and other clinic staff in the PA trainings to foster support. Previous work notes that, including clinic personnel increases stakeholder appreciation and understanding of potential contributions and effective use of CHWs [28, 39]. Another strategy may be designing future PA training sessions around the health clinic context and its existing capacities, even offering sessions onsite after clinic hours [40]. However, staff training and PA promotion efforts must not impact current services or add extra burden; new CHW-led initiatives should be seamlessly integrated as an extension of the clinic [34]. Finally, ongoing efforts to observe and support CHWs are highly beneficial in developing cohesion and improving performance [41]. However, training programmes commonly give inadequate attention to ensuring high-quality, sustained supervision, leaving CHWs unsupported and isolated [40, 54].

### Credentialing and certification

CHPs in the present study voiced a need for greater legitimacy of their role. They desired formal recognition of the specialized PA training they received and their unique expertise (e.g., via credentials or certifications), largely because many community members did not respect their knowledge. While CHWs in South Africa receive certification as ancillary health care workers under the National Qualifications Framework [58], they have little other formal recognition and want clearer pathways to regular employment and professional growth [34]. In Uganda, CHWs reported that community members negatively perceived their ability to provide assistance with NCDs, believing that CHWs were only trained to address communicable diseases, sanitation, and hygiene [20]. This need for credentialing is supported by a systematic review in which none of the seven studies offered CHWs a training certification [46]. Further complicating matters, CHWs in South Africa are often seen as ‘voluntary workers’ on temporary or unstable contracts and occupying a lower status than nurses [59]. Providing training-based credentials would increase credibility, enhances self-efficacy, strengthens their influence, and is foundational to successful programme implementation [59].

This study has several strengths. It is the first to conduct an in-depth exploration of the training needs and desires of CHPs in promoting PA as a regular part of their roles. This work was supported by the CoJ Department of Health, resulting in the participation of nearly all CHPs working in Johannesburg, South Africa. Additionally, since the CoJ strives for equal representation in their CHP hiring practices, our study sample was nearly equally divided based on sex. However, the results of this study are not necessarily representative of CHWs in other parts of South Africa or other African countries. Since the focus of the study was on PA promotion, it is possible that other lifestyle behaviours, such as healthy eating or smoking cessation, may have been given greater importance if discussed. Similarly, our discussions focused on PA promotion in isolation without considering the numerous other responsibilities that CHPs are asked to navigate on a daily basis.

## Conclusions

This study provides evidence for strategies to train CHWs to serve as a viable source of primary prevention in their local communities. Our CHWs were eager to learn about and promote PA with their patients, something that is particularly important given the increasing levels of physical inactivity, obesity, and NCDs in LMICs. However, without regular, ongoing PA training and supervision, the effectiveness of CHWs PA promotion efforts are limited. Future PA training efforts need to account for the health setting context, available resources, and incorporate a team-based learning approach that includes all clinic staff. Further, strategies should be explored to provide CHWs with greater recognition (i.e., credentialing) of their knowledge and skills to enhance their status with the local community. For CHWs to be effective in NCD prevention, it is essential to provide them with the time, effort, and resources that is given to other health conditions so that they can optimally promote PA in their local communities.

## Electronic supplementary material

Below is the link to the electronic supplementary material.

Supplementary Material 1

Supplementary Material 2

Supplementary Material 3

## Data Availability

The datasets used and/or analysed during the current study are available from the corresponding author upon reasonable request.

## Abbreviations

CHPs: community health promoters
CHWs: community health workers
CoJ: City of Johannesburg
FG: focus group
HIV: human immunodeficiency virus
LMICs: low- and middle-income countries
NCDs: non-communicable diseases
PA: physical activity
