# Supplementary material for: Assessing the physical activity training needs and preferences of community health workers in South Africa
Source: BMC Public Health. 2025 Jan 10;25:120. doi: 10.1186/s12889-025-21352-z (PMC11721254; doi:10.1186/s12889-025-21352-z)
Supplement: Supplementary file 1 — Supplementary Material 1 [file 12889_2025_21352_MOESM1_ESM.docx]

**COMMUNITY HEALTH PROMOTER QUESTIONNAIRE**

By filling out this survey, I hereby confirm that I have been informed by the researchers, Mark Stoutenberg/Estelle Watson, about the nature of the study called “Development of a Physical Activity Training Programme for Community Health Promoters”. I have had sufficient opportunity to ask questions and (of my own free will) declare myself prepared to participate in the study.

| **Please tick the response that most closely ﬁts your demographics. If the choices given do not ﬁt your situation, please feel free to write in one that does**. | | | | | |
| --- | --- | --- | --- | --- | --- |
| 1. How many years have you been working as a community health worker? | 🞎 <2 years | 🞎 2-5 years | 🞎 5-10 years | 🞎 10-20 years | 🞎>20 |
| 1. What is your gender? | 🞎 Male | 🞎 Female |  |  |  |
| 1. What is your race? | 🞎 Caucasian | 🞎African | 🞎Indian | 🞎Coloured | Other: |
| 1. What is your age? | ________ years | | | | |
| 1. On a scale of 1-5 how would you rate your skills and knowledge needed to be a community health worker? | 🞎 1  Not highly skilled | 🞎2 | 🞎3  Somewhat  skilled | 🞎4 | 🞎5  Highly skilled |
| **For the following questions, physical activity refers to any activity that you do from a low intensity level, such as walking, to a high intensity level, such as playing a competitive sport.** | | | | | |
| 1. Are you familiar with guidelines on how much physical activity you should do to be healthy? | 🞎 Yes | 🞎 No |  |  |  |
| 1. How many ***minutes*** of aerobic activity should one do per week to gain health benefits? | ________ minutes / week | | | | |
| 1. How many days per week should strength/resistance exercise be performed? (such as using weights or doing squats) |  | | | | |

**Please tick the response that most closely ﬁts your feelings toward the statement given. If examples are requested, please write all those that you feel apply in the space provided.**

1. Physical activity / exercising is beneﬁcial for your health.

🞎 Strongly agree 🞎 Agree 🞎 Disagree 🞎 Strongly disagree

1. Any amount of physical activity is beneficial to health.

🞎 Strongly agree 🞎 Agree 🞎 Disagree 🞎 Strongly disagree

1. Promoting physical activity is an important part of my job as a community health worker.

🞎 Strongly agree 🞎 Agree 🞎 Disagree 🞎 Strongly disagree

1. I regularly advise my community about the benefits of physical activity

🞎 Strongly agree 🞎 Agree 🞎 Disagree 🞎 Strongly disagree

1. I have sufficient knowledge to advise people about physical activity.

🞎 Strongly agree 🞎 Agree 🞎 Disagree 🞎 Strongly disagree

1. I believe that I can help the community to increase their physical activity levels.

🞎 Strongly agree 🞎 Agree 🞎 Disagree 🞎 Strongly disagree

1. What percentage of patients do you recommend physical activity /exercise?

🞎 0% 🞎 25% 🞎 50% 🞎 75% 🞎 All my patients

1. For what reasons do you NOT recommend physical activity / exercise?
2. If yes, what types of exercise do you recommend for people in your community?
3. What percentage of your patients in the community ask about physical activity?

🞎 None 🞎 25% 🞎 50% 🞎 75% 🞎 All of them

1. Are you aware of any exercise classes or physical activity programmes in your community?

🞎 Yes 🞎 No

1. If yes, please describe them:

🞎 Never 🞎 Seldom 🞎 Often 🞎 Always

1. If yes, do you recommend people to go to any of these programmes?

**Please provide us with information regarding your training needs and experiences regarding physical activity.**

1. Have you ever received formal training in physical activity / exercise? 🞎 Yes 🞎 No
2. If yes, how long was this training?
3. Where did this training take place? Who provided this training?
4. How interested are you in receiving formal training on physical activity / exercise?

🞎 Not interested 🞎 Slightly interested 🞎 Interested 🞎 Very interested

1. How would you like to receive training in physical activity / exercise?
2. What type of things would you like to learn about in terms of physical activity / exercise?
3. How likely would you be to apply training in physical activity / exercise in your work with patients in the community setting?

🞎 Not likely 🞎 Likely 🞎 Extremely likely
