# Supplementary material for: Assessing the physical activity training needs and preferences of community health workers in South Africa
Source: BMC Public Health. 2025 Jan 10;25:120. doi: 10.1186/s12889-025-21352-z (PMC11721254; doi:10.1186/s12889-025-21352-z)
Supplement: Supplementary file 2 — Supplementary Material 2 [file 12889_2025_21352_MOESM2_ESM.docx]

**Qualitative Discussion Guide: Physical activity training needs**

**Introduction**

Hello, my name is­ _____________ and I am from Wits University. We are here today to learn about your work as community health promoters and your thoughts about promoting physical activity in the communities where you work. We want to learn the best ways that we can provide community health promoters, across South Africa, with information and training on promoting physical activity. We will use what we learn from you today, and from our discussions with other community health promoters, to design the best possible physical activity training program.

Before we start a few comments. First, participation in this project is voluntary. You may choose not to participate at any time. You can also skip any question(s) that you do not want to answer and you can leave at any time. Second, I want to make sure it is okay to tape record the session. The tape recording is just to make sure I don’t miss any of your comments, and sometimes I can't write fast enough to get everything down. No one else, outside of our research team will hear these conversations and we will keep your comments private. Does anyone have any questions?

Before we open the discussion, could I ask you to complete this questionnaire? You may have completed something similar in the past. If so, we kindly ask that you complete this one more time.

- *Pass out questionnaire*
- *Walk group through each of the questions to expedite process*

Okay, now we will move on to our group discussion. This discussion will last about 45 minutes. I am going to ask several questions and then invite you to share your thoughts and opinions. We will first ask you three warm up questions about your roles and training as community health promoters. We will then ask you three longer questions about any physical activity training you have received and how you might like to receive this information in the future. You will do most of the talking. I will be listening and learning from you. I ask that only one participant speak at a time and that everyone is given a chance to speak. There are no right or wrong answers, just different points of view. Please feel free to share your point of view even if it is different from what others have said.

**Focus Group Questions**

1. How do you see your role or responsibility as a community health promoter (CHP)?

*Prompts: What aspects of your job do you enjoy? What aspects of your job do you dislike?*

1. What training have you received as a community health promoter?

*Prompts: Describe this training. How regularly did you receive this training?*

*What was good / what did you enjoy about this training?*

*What was bad / what did you dislike about this training?*

*Describe a training session that you have enjoyed before.*

1. How would you rate your knowledge and skills that you need to be a CHP?

*Prompts: What skills do you think you are particularly good at?*

*What skills and knowledge do you think you still need?*

*Describe the ways in which you think you could become a better CHP.*

1. Not including our training today, can you describe any physical activity training that you have received thus far in your careers as community health promoters?

*Prompts: When did this occur, and how often? Who provided the training?
 How was the training delivered? What was good/bad about the training?*

1. How would you like to receive physical activity training?

*Prompts: What kinds of things would you enjoy/ want to be included with this training?
 What kinds of things would you dislike / want excluded from the training?*

1. To what extent would you say that you have the knowledge and skills to provide physical activity education to your patients/community?

*Prompts: What additional skills and knowledge would be helpful to promote physical
 activity in your community?*

*What are your current barriers to promoting physical activity in community?*

*What physical activity information would you like to give your patients?*

*What kind of questions do you get asked that you feel you cannot answer?*

1. Is there anything else you would like to say/discuss/elaborate on the topic of physical activity training?
