# Supplementary material for: Assessing the physical activity training needs and preferences of community health workers in South Africa
Source: BMC Public Health. 2025 Jan 10;25:120. doi: 10.1186/s12889-025-21352-z (PMC11721254; doi:10.1186/s12889-025-21352-z)
Supplement: Supplementary file 3 — Supplementary Material 3 [file 12889_2025_21352_MOESM3_ESM.docx]

**Supplemental Table 1. Qualitative themes and supporting quotes.**

| **Themes** | **Categories** | **Supporting Quotes** |
| --- | --- | --- |
| **CHP roles and responsibilities in health promotion and disease prevention** | *Role as a Community Health Promoter* | - 1. “I think we are like a bridge, there is government which sets policies and standards then there are people who have to implement those things and then there are the end results, which is the citizens. Like, if they say the nurses must go and immunize the kids at schools we are the ones who have to give information to the citizens and say that on this day there will be immunization at certain points so we have to mobilize the community to go there in numbers with their kids. But now if there is an outbreak, we have to go back to the community again, if there is something wrong within the community, let’s say, like as the deputy or director indicated that our roles cuts across we have to go back to the government to say that they are saying there something wrong which is danger in in the community and so it can bring health hazards to the community.” (FG01)   2. “I wanted to say our role as health community promoters is to be outside in order to prevent illnesses. Err! Our role is we should be outside more than being inside because when we are at the clinic people are already sick. People that are at the clinic are already there for something. So our role is preventative, so we really need to be outside, prevent illnesses, to start exercising class and to speak to the community about how can we prevent them from coming to the clinic. How can we prevent, like for instance, minor illnesses like your flu, there are things that you can do that can prevent it.” (FG01)   3. “Our job is to do door to door and know our community and know their problems, and know their treatment and come back and take their EDC cards and see how many children are defaulting.” (FG03)   4. “But as a health promoter we are there to show them the importance of taking the treatment of taking the medication.” (FG05) |
|  | *Job Satisfaction and Challenges* | 1. “CHP: First things first, I love the fact that I make a whole lot of impact.   INTERVIEWER: Impact?  CHP: Yes, impacts to individuals err maybe my fellow colleagues might not realize it but we make a whole lot of impact in the community. I remember on Thursday when I was talking about us having the walks and all that, this other two old men, they were actually saying that they’re interested in coming in for the walks and all because the doctor all the time when he comes he complains about their high blood, and their high blood is always high. So the impact and the influence that we are putting in these people’s lives is very very, its real, its real and for the fact that I’m changing someone’s live it makes me feel great about myself.” (FG10)   1. “I think I will go with my colleagues because some of our facility managers they do not understand or know the scope of health promotion. That is why sometimes it’s difficult for them to say okay, you are doing this, you are doing this it’s were you end because sometimes we end up do some other things that are not within our scope of health promotion you see? That is the only thing that I might say I not like about, I do not like about my job it’s because some facility managers they sometimes give you work that is totally of irrelevant of your work.” (FG07) 2. “We have to rely on the sponsors or donations which you might or might not get. If you don’t get them, you have to cancel your event. If you get them you are lucky. So that’s one of the main challenges we are having. If maybe there could be some form of a budget for health promotion, I think things would be much better that way.” (FG05) 3. “And to help on the dislikes of health promotion. So when we starting with a health project or some sort. And then people are expecting to add, they expecting to be paid. Do you understand, like there is nothing freely. Education, yes it’s there but at the end of the day they are expecting something yeah, that’s it.” (FG05) |
| **Ongoing training to maintain skills and knowledge** | *Previous training experience* | 1. “We receive training about diseases. Ok diabetes, high blood pressure, HIV, TB, measles, polio, immunization, cancer, breast cancer, testicular cancer, prostate cancer and HPV.” (FG01) 2. “But the challenge with most of the training is that, they are certificate-based training, they are not accredited training, that you can actually use on your personal life or after health promotion”. (FG02) 3. “It depends on the facilitator or facilitators, some facilitators are formal, but usually the trainings they take the whole day.” (FG07) 4. “CHP: We do not have a plan that we know that this month we will be going there, we just come by. I don’t know how to say it but usually they don’t come, they don’t do training so often. INTERVIEWER: It is not regular?   CHP: It is not regularly, it will come sometimes. Even if there is something maybe like usually the outbreaks then that is when we will be having the trainings. It is not usually like there is something that we know every year we will have two to three trainings that we don’t know about it.” (FG07)   1. “But I think I need more trainings, you know, more especially when it comes to public speaking, public orientation like presentations kind of, yes, basically yeah.” (FG08) 2. “So most of the information that we have or most of the information that we teach at the clinics is mostly the information that we gathered for ourselves, yeah it mostly the information that we gathered ourselves. Like you google on your phone when you want to teach about something and you say today you want to teach about heart conditions then you have to google it and then read it then go teach about it.” (FG04) 3. “Normally, I think they [the trainers] are professionally trained from universities with all the degrees in health promotion” (FG08) |
|  | *Qualities and skills needed to be a Community Health Promoter* | 1. “Me I’m good in public speaking, that is the first thing about me, I am very good at public speaking, that is a yes for me.” (FG02) 2. “Some people Google, they Google information and they will ask you something that you really, wanting to know if you are clever or you know your story, so talking I don’t think for me it’s a problem because I have been talking and talking” (FG02) 3. “I think it’s an individual thing. Some of us we’ve been long here. Yes, I think I have enough experience to can handle whatever, because whatever it is that I don’t have knowledge on I will always refer.” (FG01) 4. “I am confident, to just like to give a talk, even though some of the questions for me is a challenge, like I give the talk about TB, somebody sitting there at the corner say uhm, I just want to know about the immunization you see so I must get out of my topic and then get in her topic immunization so you see it’s difficult because I am prepared to talk about TB but there somebody comes she wanted to know about immunization. So in theory I can say yes I am confident, maybe I can rate me on 9 out of 10.” (FG09) |
| **Need for PA training to foster healthy lifestyles in their communities** | *Modeling and Promoting Healthy Lifestyle Behaviors* | 1. “When they [trainers] talk about diet, I do not think about myself I think they talking to you. They should start with us so that we become masters with that in our homes, with our children our relatives or whatever before I can go and knock at a door and say no mom this is one, two, and three it must start with me. It must start with ourselves first, so the way they have put it is like a community thing, but we are left behind, we are trained for the community and not for ourselves. It’s like we are directing them to the road while we are watching with binoculars. It will be easy once we live the life of health promotion ourselves.” (FG03) 2. “I think we should, we should enforce to our clients is that medication by itself is not enough because they tend to really hundred percent on medication, they even sometimes ignore the diet. I think once we can just enforce it that physical activities, diet is equally important as the medication which you are taking. I think that is also a role that we must enforce.” (FG08) 3. “But remember the communities, they are so disadvantaged, like affordability firstly we can tell them what to do what to eat but most of them as you know in the locations they are not working so that is also eh sort of a blocking, it’s blocking the change that we’re supposed to see because they will be interested in doing or changing the behavior but now how do you expect a person to eat fruits and vegetables or whatsoever that is needed by the body and that person is not working, so that one employment it’s a problem.” (FG02) |
|  | *Gaps in knowledge and skills related to physical activity* | 1. I can rate myself 5/10 because I believe I still need more on physical activity more especially practical. (FG05) 2. “To me I think it can be five, because every time I think is the same exercises. And then sometimes the grannies have got heart problems, asthma and you don’t know which exercises that you can do with them. So we end up that we don’t know what to do.” (FG05) 3. “I won’t say we are so effective because we are not getting the trainings, we only got the training once, so we cannot, there is no way unless otherwise, there is no way we can be effective if we are not getting the physical trainings ourselves.” (FG04) |
| **Perceptions of previous PA training and suggestions for future trainings** | *Previous physical activity training experience* | 1. “Yeah that training helped a lot, especially after that training we practiced on the pregnant women, so it helped a lot via exercising” (FG02) 2. “The training was ok, but for me at some point I felt it was too short. We were just covering the basics, just on top for me. Because I think there was a time when they showed us some of the physical activities, and they also showed us for the pregnant women. I felt it was too short, we did not go deep into the physical activities.” (FG03) 3. “INTERVIEWER: Good. Besides that one day, have you received any other training in physical activity or exercise?   CHP: No.  INTERVIEWER: that was your first time?  Group: it was done twice.  INTERVIEWER: yea, she did it twice.  CHP: Yeah, that was the first and the last exercise training” (FG01) |
|  | *Preferences for future trainings* | 1. “It is not easy to hold a training especially like you can’t apply same exercise to different people isn’t it. So I think we should be having certain categories to say for the older people this is the kind of exercise that you can try to provide. And then for the teenagers you know, pregnant women this are the kind of exercises that you maybe can try to apply to them. Because you can’t, as health promoters you can’t be sticking to one thing only you need to go broader.” (FG04) 2. “And again I think it should be ongoing training you know, it should not be just once in a year because if it comes once in a year things change and people want something new, something that can make them more excited. That is what I can say, so the training should not just be once in a year it should be ongoing.” (FG04) 3. “But I think it will be much better if they can be with us, I think for a week.” (FG05) 4. “Yes, understand where we come from and then from there I think you guys are going to be able to plan a training that is going to be suitable for us understanding in mind while having the picture. Ok this is going to be feasible to apply this kind of training because I have being there, I know the space and everything.” (FG08) 5. “There are other people; I don’t know why are they excluding them in our trainings ward leaders. Because they are there every day in communities sometimes we are with them but when things are organized, they are excluded. Reason being? I don’t know and even some of the community care workers the, they must be included.” (FG10) 6. “You cannot just go out there. We want specific intense training and that is accredited because sometimes they ask if we are accredited. (FG06) 7. “CHP-1: We must be notified.   CHP-2 “And we come with the proper gear.” (FG03)   1. “And other thing, I think that if we can get to know more people who are professional in doing physical activity or exercises maybe once a month and invite them to tell us which exercises are good and which are bad that will also help.” (FG05) |
| **Application of physical activity training** | *Application of physical activity* | 1. “So I always advise them that the cleaning, I mean the cleaning, doing the garden is still part of exercise, that I always encourage them to do that...” (FG02) 2. “Attitude is very much important because even the patients become impatient whilst we are still giving health education. They don’t understand when you are giving health education what are you doing. As I am speaking now, I’m in a case of a patient who attacked me when I was giving health education. Our safety is also at stake because when they see us, they see us as a problem and they attack us.” (FG05) 3. “Relevant visual aid, is one of the things I have heard the patients say because as much as we can talk for a person to actually uhm register it, they need to see it and we have irrelevant sometimes I would say visual aid.” (FG09) |
